# Psychological Distress in Responders and Nonresponders in a 5-year Follow-up Health Survey: The RIAS Study

**DOI:** 10.2188/jea.JE20200617

**Published:** 2022-12-05

**Authors:** Megumi Tsubota-Utsugi, Yuki Yonekura, Ruriko Suzuki, Ryohei Sasaki, Kozo Tanno, Haruki Shimoda, Akira Ogawa, Seiichiro Kobayashi, Kiyomi Sakata

**Affiliations:** 1 Department of Hygiene and Preventive Medicine, Iwate Medical University School of Medicine, Iwate, Japan; 2 Graduate School of Nursing Science, St. Luke’s International University, Tokyo, Japan; 3 Department of Human Sciences, Center for Liberal Arts and Sciences, Iwate Medical University, Iwate, Japan; 4 Iwate Medical University School of Medicine, Iwate, Japan

**Keywords:** disaster victims, health surveys, participation, psychological distress, survivors

## Abstract

**Background:**

People with poor health or mental conditions are generally unwilling to participate in the health examinations, and no studies have directly examined the relationship of psychological distress among disaster survivors with participation status to date. The present study thus examined psychosocial differences according to the respondent status in a 5-year follow-up survey among participants in the prospective health surveys on survivors of the Great East Japan Earthquake and Tsunami Disaster study in Iwate Prefecture, Japan.

**Methods:**

We analyzed data from 10,203 Japanese survivors aged ≥18 years (mean age, 65.6 years; 38.0% men) and who underwent health examinations at baseline in 2011. Participants were classified into responders and nonresponders according to their 2015 health examination participation status. Psychological distress was evaluated using the Kessler 6 scale and categorized as none, mild, and severe. Multinominal logistic regression was used to examine the risk of psychological distress in relation to participation status.

**Results:**

In the 2015 survey, 6,334 of 6,492 responders and 1,686 of 3,356 nonresponders were analyzed. The most common reasons for nonparticipation in the survey were participated in other health examinations, examined at a hospital, and did not have time to participate. Nonresponse in males was associated only with mild psychological stress, whereas nonresponse in females was associated with mild and severe psychological distress.

**Conclusion:**

Nonresponders in the follow-up survey had a higher risk of psychological distress than responders. Continuous monitoring of the health of nonresponders and responders may help to prevent future health deterioration.

## INTRODUCTION

In March of 2011, a massive earthquake and huge tsunami caused immense damage in eastern Japan. By the end of 2015, more than 15,000 deaths were confirmed, and 2,558 people were still missing.^1^^,^^2^ Since 6 months after the disaster, the Research Project for Prospective Investigation of Health Problems Among Survivors of the Great East Japan Earthquake and Tsunami Disaster (RIAS) has provided free annual health examinations to all survivors in Iwate Prefecture who wish to participate.^3^^,^^4^

Psychological distress is an important indicator in longitudinal studies to better understand the risk of further health deterioration^5^^,^^6^ such as insomnia,^7^^,^^8^ premature mortality,^9^ and maladjustment to environmental changes after a disaster.^10^^,^^11^ In the 5 years since the Great East Japan Earthquake (GEJE), the number of participants in RIAS has varied each year. At the baseline survey in 2011–2012, a total of 10,203 people participated in the survey. The number of participants decreased over time, from 7,275 people in 2012, to 6,878 people in 2013, 6,581 people in 2014, and 6,492 people in 2015. Survivors with serious psychological and/or physical health problems may be reluctant to undergo health examinations and surveys. Given the evidence that nonparticipants in health examinations conducted by municipalities are more likely to have unhealthy lifestyles and poorer health than participants,^12^^–^^14^ which could lead to severe bias,^15^ gathering the characteristics of survivors who did not participate in the follow-up study is considered for preventing health deterioration and providing early treatment or support to survivors of disasters.

The present study examined the difference in psychological distress and characteristics by participation status after the 5-year follow-up survey of survivors of the GEJE in Iwate Prefecture, Japan. Also, we described the distribution of the reasons for non-participation.

## METHODS

### Study design

The details of the RIAS study have been reported previously.^4^^,^^16^ In brief, the survey was conducted as part of the national health examination system based on the Regulation Act on Assurance of Medical Care for Elderly People of 1982. This survey employed a common questionnaire inquiring about health conditions and lifestyles to people residing in Japan who do not have access to other health examinations, such as those provided by their workplace.^17^ In RIAS, both individuals eligible for the original health examination program and ineligible individuals who wish to participate in the survey were permitted to receive annual health examinations. Baseline data collection was conducted between September 2011 (ie, 6 months after the disaster) and February 2012 for all residents aged ≥18 years in Yamada town, Otsuchi town, and Rikuzentakata city in Iwate Prefecture, which were all heavily damaged by the GEJE. Follow-up surveys were repeated annually using similar methods. The baseline data, data from the follow-up survey in 2015, and data from an additional complementary questionnaire survey for non-participants of the follow-up survey in 2015 were used for this study. After the annual follow-up survey in 2015 for each study area, we identified subjects who did not participate in the follow-up survey. Then, we sent the additional complementary questionnaires to them. The questionnaires were collected by mail or by investigator’s visit. The investigators visited the subjects at least twice if the response had not been sent by mail and the subjects were absent.

**Figure 1.**
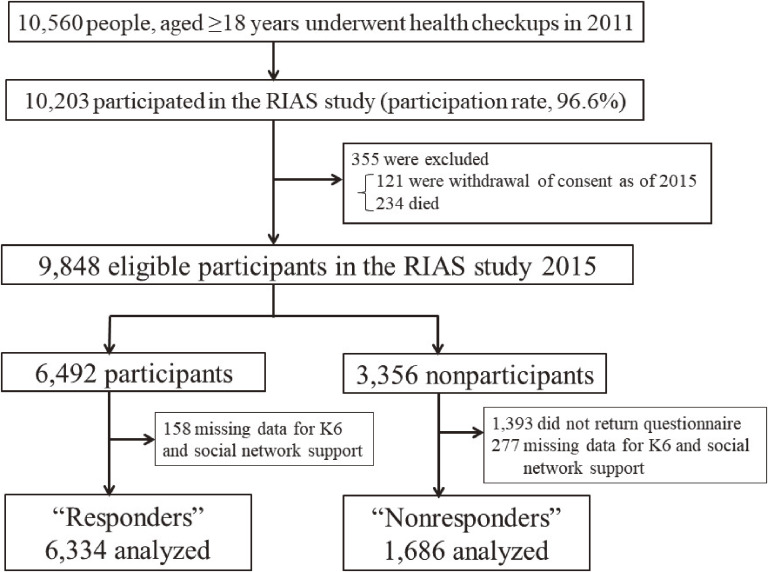
Flowchart depicting responders and nonresponders in the Research Project for Prospective Investigation of Health Problems Among Survivors of the Great East Japan Earthquake

Figure 1 presents the flow diagram of the present study. In total, 10,560 individuals aged ≥18 years underwent the initial baseline health examination, and 10,203 individuals provided written informed consent to participate in the RIAS study (96.6% participation rate). During the 5-year follow-up period, 355 participants were excluded from the follow-up analyses because consent to participate was withdrawn (*n* = 121) or they died (*n* = 234), resulting in the inclusion of 9,848 participants (mean age, 61.0 years; 39.0% men). Of the eligible participants in 2015, 6,492 (65.9%) participated in the annual survey and completed the RIAS questionnaire (responders). A complementary questionnaire was sent to the eligible participants who had not returned the questionnaire by October 2015 (nonresponders). In the study, 6,334 of 6,492 responders and 1,686 of 3,356 nonresponders were analyzed.

This noninvasive observational study complied with the Declaration of Helsinki, and its protocol was approved by the Institutional Review Board of Iwate Medical University (approval reference number: H23-69). Participants were informed of the potential risks and benefits of the study, and all subjects provided written informed consent.

### Measures

Demographic, lifestyle, and health variables, including psychological and physical factors, were assessed annually using multiple self-administered questionnaires and anthropometric measurements among RIAS participants. The questionnaire distributed at the annual survey consisted of the following details in sequence: the severity of damage, living status, history of diseases, lifestyle characteristics including smoking, drinking, diet,^16^^,^^18^ and physical activity,^19^ psychological distress,^20^^–^^22^ insomnia,^23^^,^^24^ and social support networks.^25^^,^^26^ Conversely, the complementary questionnaire submitted to nonresponders of the 2015 survey only assessed the following variables; demographic variables (age, damage attributable to the GEJE such as housing damage, residential status, death of co-residing family, and marital status), reasons for not participating in the 2015 survey, and health variables (psychological distress and insomnia, sleep medication use, and social support networks). In this study, we used the variables included in the 2015 survey because the items in the complementary questionnaire were the same as those in RIAS. For other variables, especially regarding lifestyles, we used data from the baseline survey.

### Psychological distress

Psychological distress was assessed using the six-item Kessler scale (K6).^20^^–^^22^ The K6 is composed of six questions regarding how often an individual has experienced the following emotions in the previous month: nervousness, hopelessness, restlessness, extreme sadness, exhaustion, and worthlessness. The responses range from none of the time (0 points) to all of the time (4 points), and the total score is designated as the sum of scores for the six items. Thus, the score ranged 0–24, and psychological distress was graded as follows: severe, ≥13; mild, 5–12; and none, 0–4.^20^^,^^27^^,^^28^

### Other characteristics

Information regarding the severity of housing damage was assessed by asking the participants to respond to the following question: “What was the degree of housing damage due to the disaster?” The potential answers were as follows: completely destroyed, large-scale damage, small-to-moderate damage, partly damaged, not destroyed but flooded, and not destroyed or flooded. On the basis of these answers, the participants were categorized into three groups: extensive (completely destroyed and large-scale damage), partial (small-to-moderate damage, partly damaged, and not destroyed but flooded), and no damage (not destroyed or flooded). The current residential status was categorized into unchanged, temporary housing, and others based on the following question: “Which residence do you mainly live in now?” As the query on residential status changed according to the stage of the disaster, the responses obtained in 2015 were as follows: own home, home of a relative or acquaintance, prefabricated temporary housing, private temporary housing, rented accommodation, a new home reconstructed in the same location, a new home reconstructed in a different location, or other residence.

Other factors, including body mass index (BMI; weight in kg/height in m^2^), hypertension, diabetes mellitus, and hypercholesterolemia were also measured via anthropometric measurements at the baseline health examinations. Each participant was classified by BMI as underweight (<18.5 kg/m^2^), normal weight (18.5–24.9 kg/m^2^), or overweight (≥25.0 kg/m^2^). Blood pressure was measured twice consecutively in a sitting position using an automatic device after urination and a 5-min rest period. Hypertension was defined as blood pressure ≥140/90 mm Hg and/or the self-reporting of hypertension treatment. Diabetes mellitus was diagnosed as a random blood glucose level ≥11.11 mmol/L (≥200 mg/dL), a glycosylated hemoglobin level ≥6.5% (NGSP),^29^ and/or the self-reporting of diabetes treatment. Hypercholesterolemia was defined as total cholesterol levels of ≥5.68 mmol/L (≥220 mg/dL) and/or the self-reporting hypercholesterolemia treatment.

### Statistical analysis

Because the results of previous studies^4^^,^^30^^,^^31^ suggested that the association between participation in the examination and psychological distress is different by sex and because of the significant interaction between respondent status and sex (*P* < 0.001), the analysis was performed separately for men and women. The characteristics were compared between responders and nonresponders in the 2015 follow-up survey using Student’s *t*-test for continuous variables and the chi-squared test for categorical variables.

Multinomial logistic regression analysis was used to calculate the odds ratios (ORs) and 95% confidence intervals (CIs) for the response status after adjustment for other putative confounding factors according to psychological distress (severe/mild vs none psychological distress as a reference). The potential confounding factors were selected from variables that were suggested to be associated with both mental health and participation in health examination by previous studies,^4^^,^^10^^,^^11^^,^^30^^,^^31^ and/or variables with an age-adjusted *P* ≤ 0.20.

Covariates included in the final model were as follows: the number of times participating in the survey (1–4 times), the severity of housing damage (no damage, partial, extensive, or missing), residential status (unchanged, temporary housing, others, or missing), death of co-residing family (yes, no, or missing), marital status (unmarried, married, divorced, widowed, or missing), insomnia (yes, no, or missing), history of stroke (presence or absence), hypertension (presence or absence), current smoking (yes or no), current drinking (yes or no), physical activity (<23 metabolic equivalents (METs)·h/week or ≥23 METs·h/week), and social support networks (<12 or ≥12) for men; and the number of times participating in the survey (1–4 times), city of residence (Yamada, Ohtsuchi, or Rikuzentakata), the severity of housing damage, residential status, death of co-residing family, marital status, insomnia, sleep medication use (yes, no, or missing), current smoking, and social support networks in women. Possible interactions were tested by introducing a multiplicative term into the main effect models. A subgroup analysis was further performed to evaluate whether the association with the risk of psychological distress was attenuated or reversed by age groups (<65 and ≥65 years). We calculated the Variance Inflation Factors (VIF) for each explanatory variable to assess the multicollinearity. The VIFs ranged from 1.03 to 2.45 for males and from 1.02 to 2.40 for females. Thus, we concluded that no multicollinearity was present in our multivariate analysis. We also calculated events per variables (EPV) of each model to assess the risk of inaccurate parameter estimation. EPVs greater than 10 are high enough for the estimation performance.^32^ The EPVs in our logistic regression models ranged from 18.8 to 125.2, indicating no serious problems in the parameter estimation. Multiple imputations were applied using 20 imputed datasets generated with a multiple imputation approach using PROC MI and PROC MIANALYZE to ensure that missing data did not introduce bias regarding the association between psychological distress and participation status. This analysis was performed assuming that data were missing at random,^33^^,^^34^ All statistical analyses were performed using SAS, version 9.4 (SAS Institute, Inc., Cary, NC, USA). For all analyses, statistical significance was defined as α < 0.05 for two-sided tests.

## RESULTS

The characteristics of the responders and nonresponders are shown by sex in Table 1. Severe psychological distress was observed among 1.8% of male and 3.0% female responders, respectively, compared with 3.9% and 6.2% of male and female nonresponders, respectively. Conversely, the rates of mild psychological distress in male and female responders were 17.7% and 25.3% versus 28.3% and 31.2%, respectively, of male and female nonresponders. Comparison of the characteristics by the respondent status enrolled in the 2015 survey by sex is presented in Table 1. Responders were more likely to report the death of someone living in the same residence in the GEJE. Nonresponders also had a higher prevalence of psychological distress and weak social networks. Current smoking was more prevalent among nonresponders, whereas current drinking was more frequent only among female nonresponders in the 2015 survey.

**Table 1.** Differences in characteristics between responders and nonresponders in the 2015 follow-up survey (*n* = 8,020)

	Survey year	All participants	Females (*n* = 4,989)			Males (*n* = 3,031)			*P* ^b^
	
			Responders	Nonresponders	*P* ^a^	Responders	Nonresponders	*P* ^a^	
Number of study subjects		8,020	3,993	996		2,341	690		
Number of times participating, mean (SD)		4.1 (1.4)	4.6 (0.8)	1.9 (1.0)		4.6 (0.8)	1.8 (1.0)		
Age, mean (SD)	2015	65.6 (13.6)	65.9 (12.4)	60.0 (17.2)	*<0.001*	66.8 (12.4)	60.9 (16.7)	*<0.001*	*<0.001*
Age ≥65 years, %	2015	62.3	63.6	42.7	*<0.001*	72.5	48.8	*<0.001*	0.142
Residence, %	2015				*<0.001*			*0.194*	*0.981*
Yamada		31.7	29.8	35.3		33.2	32.0		
Ohtsuchi		20.5	20.2	23.7		19.1	22.2		
Rikuzentakata	2015	47.8	50.0	41.0		47.7	45.8		
Severity of housing damage, %	2015				*<0.001*			*<0.001*	*<0.001*
No damage		41.5	42.9	40.8		40.2	38.6		
Partial		18.1	17.0	19.3		18.8	20.4		
Extensive		38.2	38.9	34.0		39.9	33.8		
Missing		2.3	1.2	5.9		1.1	7.3		
Residential status, %	2015				*<0.001*			0.003	*<0.001*
Unchanged		59.0	58.8	60.3		58.4	60.4		
Temporary housing		21.1	21.9	18.0		21.8	18.8		
Others		19.3	19.0	19.8		19.5	19.3		
Missing		0.7	0.4	1.9		0.3	1.5		
Death of co-residing family, %	2015	12.0	8.8	22.9	*<0.001*	9.4	24.1	*<0.001*	*<0.001*
Missing		0.1	0.1	0.2		0.1	0.0		
Psychological distress, %	2015				*<0.001*			*<0.001*	*<0.001*
None		72.8	71.7	62.6		80.5	67.8		
Mild		24.1	25.3	31.2		17.7	28.3		
Severe		3.1	3.0	6.2		1.8	3.9		
Marital status, %	2015				*<0.001*			*<0.001*	*<0.001*
Unmarried		8.6	5.4	8.9		10.9	19.3		
Married		70.2	68.0	62.3		77.7	69.1		
Divorced		4.0	3.9	6.6		3.0	4.5		
Widowed		16.8	22.6	21.2		8.2	6.4		
Missing		0.3	0.1	1.0		0.3	0.7		
Insomnia, %	2015	32.4	38.3	32.9	*0.003*	24.4	25.2	*0.827*	*<0.001*
Missing		1.4	1.8	1.3		1.1	0.9		
Sleep medication use, %	2015	14.1	17.9	16.1	*<0.001*	8.3	8.7	*<0.001*	*<0.001*
Unknown		0.4	0.1	1.3		0.1	1.6		
Weak social networks, %	2015	24.7	22.3	29.0	*<0.001*	24.6	33.0	*<0.001*	*<0.001*
BMI, %	2011				*<0.001*			*0.002*	*0.239*
<18.5 kg/m^2^		2.9	3.4	6.1		1.1	1.7		
18.5–24.9 kg/m^2^		64.8	67.6	63.9		63.2	55.9		
≥25.0 kg/m^2^		32.3	29.0	30.0		35.8	42.3		
History of stroke, %	2011	3.6	2.7	2.9	*0.754*	4.8	5.1	*0.757*	*0.603*
History of myocardial infarction, %	2011	0.8	0.4	0.6	*0.261*	1.2	2.5	*0.021*	*0.990*
Hypertension, %	2011	44.2	41.6	38.6	*0.083*	50.6	45.9	*0.031*	*0.790*
Diabetes mellitus, %	2011	9.0	6.8	6.6	*0.813*	12.8	11.6	*0.411*	*0.115*
Hypercholesterolemia, %	2011	29.2	32.2	25.8	*<0.001*	26.1	27.3	*0.563*	*0.376*
Current smokers, %	2011	14.9	5.0	11.2	*<0.001*	26.7	36.8	*<0.001*	*0.395*
Current drinkers, %	2011	18.5	3.7	6.6	*<0.001*	42.5	40.0	*0.242*	*0.099*
Physical inactivity (<23 METs·h/week), %	2011	64.6	68.0	64.6	*0.092*	60.0	60.7	*0.282*	*0.399*
Missing		0.7	64.6	0.6		0.6	0.1		

BMI, body mass index; MET, metabolic equivalents; SD, standard deviation.

^a^Obtained using Student’s *t*-test for continuous variables and the chi-squared test for categorical variables, comparing nonresponders with responders.

^b^Obtained using Student’s *t*-test for continuous variables and the chi-squared for categorical variables, comparing males with females.

eTable 1 compares the baseline characteristics of responders and nonresponders among follow-up eligible participants of RIAS. Many nonresponders who did complete the complimentary survey lived with temporary housing, moved multiple times after the earthquake, and displayed a higher prevalence of psychological distress and insomnia at the initial survey. These characteristics were also observed among responders who did not answer questions regarding physical distress and social support, illustrating that people who experienced severe damage because of the disaster were reluctant to answer psychosocial questions. Meanwhile, nonresponders who did not complete the complementary questionnaire were younger than their counterparts.

The most common reasons provided for nonparticipation among nonresponders in the 2015 survey are outlined in Table 2. As a whole, many nonresponders reported that they participated in other health examinations, they received an examination at a hospital, or they did not have time to participate. Other reasons included gestation, an unwillingness to participate, providing nursing care to family members, work obligations, regularly seeing a doctor, difficulty walking, no means of transportation, or unknown reasons. The distribution of reasons for no participation was similar in both sex. On the other hand, the distribution of reasons for no participation was different by age groups. Most common reason among those aged <65 years was “Participated in other health examinations” (55.8%); the second most common reason was “Did not have time to participate”. In contrast, most common reason in elderly people (aged ≥65 years) was “Examined at a hospital” and “Unable because of illness”; other reason(s), which include that they cannot walk or they need assistance to go to health examination site, were more common in elderly (7.9% vs 2.0% and 20.6% vs 8.4%, respectively) than younger people.

**Table 2.** Reasons for non-participation among participants who did not respond in the 2015 survey^a^ (*n* = 1,686)

	Total (*n* = 1,686)	By sex		By age-group	
	
		Male (*n* = 690)	Female (*n* = 996)	<65 years (*n* = 924)	≥65 years (*n* = 762)
Participated in other health examinations	34.8	35.7	34.1	55.8	9.2
Examined at a hospital	28.8	29.1	28.6	10.7	50.8
Underwent a complete medical examination	5.8	7.3	4.7	4.3	7.5
Did not have time to participate	22.7	21.2	23.8	30.4	13.4
Inconvenient location	2.0	1.0	2.7	0.4	3.9
Unknown or forgot	2.6	4.5	1.3	1.3	4.2
Did not desire to undergo the examination	4.2	5.1	3.6	3.1	5.5
Unable because of illness	4.6	3.2	5.6	2.0	7.9
Others	13.9	13.8	14.1	8.4	20.6

Data are presented as percentages.

^a^Multiple responses were allowed for this question.

The multinominal logistic regression results are presented in Table 3. A higher risk of psychological distress was observed among female nonresponders. Compared with the findings in female responders, nonresponse among women was positively and significantly associated with mild and severe psychological distress. For male participants, nonresponse was significantly associated with mild psychological distress, whereas no significant association with severe psychological distress was noted although there was a significant trend for an increased risk of psychological distress among nonresponders (*P* for trend = 0.0019). Complete-case analyses performed with only those participants who answered all questionnaires did not alter our results; nonresponse among women (*n* = 4,729) was positively and significantly associated with mild (OR 1.46; 95% CI, 1.16–1.85) and severe psychological distress (OR 2.42; 95% CI, 1.48–3.96), and nonresponse among men (*n* = 2,873) was only associated with mild psychological distress (OR 1.77; 95% CI, 1.30–2.42; data not tabulated).

**Table 3.** Summary of the associations of psychological distress with participation status among survivors

Variable	Psychological distress (K6)			*P* for interaction

	None (0–4)	Mild (5–12)	Severe (13–24)	
**Male (*n* = 3,031)**				
Number of participants	2,352	610	69	
Nonresponders	468	195	27	
**Age-adjusted**	Reference	1.85 (1.51–2.26)	2.41 (1.45–4.00)	
**Multivariate-adjusted^a^**	Reference	1.69 (1.26–2.26)	1.66 (0.76–3.62)	
***P* for trend**	0.0019			
By age				*0.608*
<65 years (*n* = 998)	Reference	1.31 (0.81–2.10)	1.20 (0.49–2.94)	
≥65 years (*n* = 2,033)	Reference	1.91 (1.31–2.79)	1.99 (0.58–6.88)	

**Female (*n* = 4,989)**				
Number of participants	2,864	1,009	120	
Nonresponders	623	311	62	
**Age-adjusted**	Reference	1.43 (1.23–1.67)	2.32 (1.68–3.22)	
**Multivariate-adjusted^a^**	Reference	1.46 (1.17–1.84)	2.30 (1.45–3.64)	
***P* for trend**	<0.0001			
By age				*0.051*
<65 years (*n* = 2,026)	Reference	1.42 (1.03–1.94)	1.88 (0.92–3.81)	
≥65 years (*n* = 2,963)	Reference	1.53 (1.10–2.12)	2.68 (1.34–5.34)	

^a^Adjusted for age plus variables with age-adjusted *P* ≤ 0.20, and the independence of the variables relative to each of the other variables in the prediction psychological distress risk was tested.

In this study, there were no significant interactions of respondent status with age group and social support network (*P* > 0.50), whereas marginal significant heterogeneity between those aged <65 and ≥65 years was a risk for psychological distress in women. Subgroup analysis by age (<65 and ≥65 years) showed similar results in men and women aged ≥65 years as those for the entire study cohort.

As a sub-analysis, the differences in the association between no participation and psychological distress according to various reasons were examined (eTable 2). Among male nonresponders, no significant difference in association with psychological distress by reasons was found. Among females, nonresponders who did not participate in the examination because of “Unknown for forgot” were more likely to have mild psychological distress (OR 4.55; 95% CI, 1.04–19.87). Also, female nonresponders with the reason “Unable because of illness” were more likely to have mild or severe psychological distress than females without the reason (OR 2.16; 95% CI, 1.04–4.45 and OR 6.91; 95% CI, 2.23–21.40, respectively).

## DISCUSSION

In this observational health study of survivors of the GEJE, we found that nonresponse in the follow-up survey 5 years after the disaster was significantly associated with an increased risk of psychological distress. This is the first study to describe psychological distress among nonresponders in the follow-up survey, which has been rarely addressed, but it may represent important evidence supporting the need for a longitudinal study to assess the long-term health of survivors.

The reasons for nonparticipation in the general health survey were described in previous studies, and the associations of an unhealthy lifestyle, worsening health,^12^^–^^14^^,^^35^ and work responsibilities^15^ with nonparticipation were consistent with our results. Meanwhile, the higher rates of current smoking and drinking among nonresponders may reflect that younger people failed to participate. Conversely, our findings that the risk of severe psychological or mental disorders was higher among subjects who completed the questionnaire without answering psychosocial questions. This could be explained by the fact that psychosocial questions are sensitive in nature, thereby triggering nonresponse.^35^

The present study also indicated that the risk of psychological distress among nonparticipants is different among age groups. Individuals aged ≥65 years had a higher risk of mild psychological distress in both sex, and women aged ≥65 years had a two-fold higher risk than younger females. A part of this result can be explained by the reason for not having the health examination. For those aged <65 years, the reason for not participating in the health examination was that they had a medical examination at work or experienced inconvenience, which is not directly related to their health condition. Conversely, lack of participation among those aged ≥65 years was related to their condition, such as that they were in poor physical condition, could not walk, and needed assistance. These results can explain that among younger people, unexamined patients were not necessarily related to poor mental health, and among elderly people, psychological distress was higher in unexamined patients. The level of social support is also an important factor that explains this result. Social support is a well-known protective factor for mental health,^36^ and it promotes health examination visit.^30^^,^^31^ Because the relationship between social support and participation in health examination is stronger in men,^30^^,^^31^ the relationship between nonparticipation and greater psychological distress in men may have become weaker in the multivariate model with social support. The sub-analysis indicated that female nonresponders who did not participate in the examination because they did not know or forgot or because of illness were more likely to have mild or severe psychological distress. This result and the difference in distribution of the reason by age groups also support the above mentioned interpretation of the difference by age group. Our results that psychological distress was more common among women than among men agreed with previous findings.^4^ Suicide risk associated with mental disorders including depression is generally two-fold higher among men than among women.^37^^,^^38^ Furthermore, some studies reported that post-disaster suicide risks were higher among men than among women,^39^^,^^40^ which may reflect the relative deficiency of mental healthcare services in disaster areas. Thus, women may possess coping skills to better prevent aggravation than men despite having a higher prevalence of psychological distress, but such factors were not clarified in the present study.

Several limitations must be considered when interpreting our findings. First, because we collected data from participants who were willing to participate in the RIAS, the sample of the present study may not faithfully represent all survivors in the disaster area. In particular, because our study’s target population lived in a disaster area and experienced a disaster, individuals with serious psychological problems, physical health problems, or both may not have participated in the health examinations in 2011. In fact, the proportion of people with psychological distress among all participants in the present study in 2015 was not different from the proportion of the general population in Japan.^41^ This may have led to underestimation of the effects of non-participation on psychological stress. Future studies are expected to examine the health characteristics of people who did not initially participate in the survey. Similarly, although the response rate to our complementary survey was relatively high, about 50% of the nonresponders in the RIAS study were not included in the analysis because of nonresponse to the survey or missing data. As mentioned above, people who did not respond to the survey or people with missing data may have been more likely to have health problems. This could lead to underestimation of the association between non-participation and psychological distress. Second, it is possible that survivors tended to show higher K6 score despite the less severe latent distress. Mental disorders become more prevalent after a disaster,^42^^,^^43^ and there is the possibility that mental health issues were over-reported. Similarly, increased responses in mild psychological distress can lead to deteriorating reliability. Third, due to the nature of the design of this study, the causal relationship between nonparticipation and psychological distress is unclear. Also, the lifestyle variables at baseline that we used in this study may have changed during the period. Finally, the assumption of missingness at random may not be true for our multiple imputation procedure for handling missing data, however, our complete-case analysis using participants without any missing data replicated the same association between the response status and psychological distress, suggesting that our results were robust.

In conclusion, nonresponders in the follow-up survey had a higher risk of psychological distress than responders, with a higher impact among female than male and among older individuals than younger individuals. The RIAS survey will continue to be performed annually through 2021; thus, the length of the follow-up period may be considered as a strength of this study. Psychological distress indicators in the longitudinal study reflecting all survivors are essential for establishing the requirements for interventions that would prevent and treat psychological and physical health after massive disasters. In parallel with efforts to maintain the participation rate and enhance the health services provided to survivors, continuous monitoring of the health both nonresponders and responders in the follow-up survey may help prevent future health deterioration from a public health perspective.

## SUPPLEMENTARY MATERIAL

eTable 1. Differences in baseline characteristics among follow-up eligible subjects: The RIAS (n = 9,848)

eTable 2. Multivariate-adjusted^a^ odds ratio (95% confidence interval) of psychological distress with reasons for non-participation among nonresponders
