# Supplementary material for: Psychological Distress in Responders and Nonresponders in a 5-year Follow-up Health Survey: The RIAS Study
Source: J Epidemiol. 2022 Dec 5;32(12):527–34. doi: 10.2188/jea.JE20200617 (PMC9643786; doi:10.2188/jea.JE20200617)
Supplement: Supplementary file 1 [file je-32-527-s001.pdf]

eTable 1. Differences in baseline characteristics among follow-up eligible subjects: The RIAS (n=9,848)

	Responders		Nonresponders			P^a	P^b
	Analyzed	Excluded	Analyzed	Excluded	Not returned		
Number of study subjects	6,334	158	1,686	277	1,393		
Sex, male %	37.0	34.8	40.9	39.7	42.1	*<0.001*	*<0.001*
Age, mean (SD)	62.4 (12.4)	68.5 (9.6)	56.5 (16.7)	67.5 (12.9)	55.9 (48.6)		*<0.001*
Age, ≥65 years %	49.2	69.0	35.2	67.5	38.5	*<0.001*	*<0.001*
Residence, %						*0.377*	*<0.001*
Yamada	31.1	50.6	34.0	38.6	25.5		
Ohtsuchi	19.8	30.4	23.1	14.8	20.2		
Rikuzentakata	49.2	19.0	42.9	46.6	50.3		
Relocation after the disaster, time(s) %						*0.740*	*<0.001*
0	48.3	43.7	50.8	58.8	40.6		
1	29.6	31.7	29.4	26.0	31.1		
≥2	21.5	24.7	19.2	15.2	27.4		
Missing	0.6	0.0	0.6	0.0	0.9		
Residential status, %						*0.033*	*<0.001*
Temporary housing	23.4	20.9	20.9	19.1	26.9		
Others	75.2	77.2	76.3	79.4	71.7		
Missing	1.4	1.9	2.9	1.4	1.4	*0.018*	*<0.001*
Psychological distress, %						*0.018*	*0.003*

None	56.8	48.7	57.3	60.7	52.4		
Mild	36.0	38.6	34.5	32.9	38.4		
Severe	5.5	9.5	6.9	5.1	7.7		
Missing	1.8	3.2	1.3	1.4	1.5		
Insomnia, %	33.1	41.1	29.8	31.1	33.0	*0.086*	*0.016*
Missing	1.5	1.3	1.1	0.4	1.9		
Weak social networks, %	39.6	43.7	41.9	44.8	42.6	*0.031*	*0.067*
Missing	2.0	4.4	2.0	2.5	2.0		
Body mass index, %						*<0.001*	*<0.001*
<18.5 kg/m^2	2.5	1.3	4.3	3.6	3.9		
18.5–24.9 kg/m^2	66.0	62.7	60.6	57.8	62.7		
≥25.0 kg/m^2	31.5	36.1	35.1	38.6	33.4		
History of stroke, %	3.5	5.1	3.8	7.6	2.7	*0.727*	*0.002*
History of myocardial infarction, %	0.7	1.3	1.4	1.4	1.0	*0.008*	*0.060*
Hypertension, %	44.9	53.4	41.6	56.7	39.1	*0.002*	*<0.001*
Diabetes mellitus, %	9.0	10.1	8.7	11.9	10.1	*0.466*	*0.330*
Hypercholesterolemia, %	30.0	30.4	26.4	30.0	28.4	*0.011*	*0.064*
Current smokers, %	13.0	10.1	21.7	15.2	26.1	*<0.001*	*<0.001*
Current drinkers, %	18.1	13.9	20.3	18.1	19.0	*0.115*	*0.019*
Physical inactivity (<23 METs·h/week), %	65.0	70.9	63.0	63.5	64.4	*0.082*	*0.302*

Missing	0.7	1.3	0.4	0.7	0.5

BMI, body mass index; SD, standard deviation.

[a.] Obtained using Student's t-test for continuous variables and the chi-squared test for categorical variables, comparing nonresponders with responders.

[b.] Obtained using one-way analysis of variance for continuous variables and the chi-squared test for categorical variables, comparing responders-analyzed excluded; and nonresponders-analyzed excluded and not returned.

eTable 2. Multivariate-adjusted[a] odds ratio (95% confidence interval) of psychological distress with reasons for non-participation among nonresponders

Variable	Psychological distress (K6)		
	None (0–4)	Mild (5–12)	Severe (13–24)
**Male (n=690)**			
Number of nonresponders	468	195	27
Participated in other health examinations	Reference	0.92 (0.49–1.71)	0.65 (0.18–2.43)
Examined at a hospital	Reference	1.50 (0.84–2.67)	1.90 (0.64–5.68)
Underwent a complete medical examination	Reference	0.59 (0.25–1.40)	NE
Did not have time to participate	Reference	0.85 (0.45–1.58)	0.98 (0.28–3.43)
Inconvenient location	Reference	3.09 (0.43–22.40)	NE
Unknown or forgot	Reference	1.63 (0.65–4.06)	3.57 (0.35–36.72)
Did not desire to undergo the examination	Reference	1.01 (0.38–2.68)	4.05 (0.86–18.96)
Unable because of illness	Reference	0.36 (0.12–1.08)	4.51 (0.43–47.57)
Others	Reference	0.92 (0.50–1.71)	1.02 (0.26–4.06)
**Female (n=996)**			
Number of nonresponders	623	311	62
Participated in other health examinations	Reference	0.92 (0.53–1.59)	1.20 (0.44–3.28)
Examined at a hospital	Reference	0.87 (0.52–1.43)	1.45 (0.60–3.47)
Underwent a complete medical examination	Reference	0.45 (0.18–1.13)	0.74 (0.11–4.85)
Did not have time to participate	Reference	1.08 (0.64–1.82)	2.33 (0.89–6.12)
Inconvenient location	Reference	0.72 (0.27–1.90)	0.50 (0.08–3.03)
Unknown or forgot	Reference	4.55 (1.04–19.87)	NE

Did not desire to undergo the examination	Reference	0.53 (0.20–1.36)	0.36 (0.02–5.33)
Unable because of illness	Reference	2.16 (1.04–4.45)	6.91 (2.23–21.40)
Others	Reference	1.12 (0.69–1.82)	1.02 (0.44–2.36)

NE, not able to estimate

[a] Adjusted for age plus variables with age-adjusted $P \leq 0.20$, and the independence of the variables relative to each of the other variables in the prediction psychological distress risk was tested.
